# Increased corticosterone in peripubertal rats leads to long-lasting alterations in social exploration and aggression

**DOI:** 10.3389/fnbeh.2013.00026

**Published:** 2013-04-04

**Authors:** Vandana Veenit, Maria I. Cordero, Stamatina Tzanoulinou, Carmen Sandi

**Affiliations:** ^1^Laboratory of Behavioral Genetics, School of Life Sciences, Brain Mind Institute, Ecole Polytechnique Fédérale de LausanneLausanne, Switzerland; ^2^Child and Adolescent Service of Psychiatry, Hospital University of GenevaGeneva, Switzerland

**Keywords:** corticosterone, puberty, adolescence, stress, sociability, aggressive behavior, emotion

## Abstract

Stress during childhood and adolescence enhances the risk of psychopathology later in life. We have previously shown that subjecting male rats to stress during the peripubertal period induces long-lasting effects on emotion and social behaviors. As corticosterone is increased by stress and known to exert important programming effects, we reasoned that increasing corticosterone might mimic the effects of peripubertal stress. To this end, we injected corticosterone (5 mg/kg) on 7 scattered days during the peripuberty period (P28-P30, P34, P36, P40, and P42), following the same experimental schedule as for stress administration in our peripubertal paradigm. We measured play behavior in the homecage and, at adulthood, the corticosterone response to novelty and behavioral responses in tests for anxiety- and depression-like behaviors, aggression, and social exploration. As compared to vehicle, corticosterone-treated animals exhibit more aggressive play behavior during adolescence, increased aggressive behavior in a resident-intruder (RI) test while reduced juvenile exploration and corticosterone reactivity at adulthood. Whereas the corticosterone treatment mimicked alterations induced by the peripuberty stress protocol in the social domain, it did not reproduce previously observed effects of peripuberty stress on increasing anxiety-like and depression-like behaviors, respectively evaluated in the elevated plus maze (EPM) and the forced swim tests. Our findings indicate that increasing corticosterone levels during peripuberty might be instrumental to program alterations in the social domain observed following stress, whereas other factors might need to be recruited for the programming of long-term changes in emotionality. Our study opens the possibility that individual differences on the degree of glucocorticoid activation during peripuberty might be central to defining differences in vulnerability to develop psychopathological disorders coursing with alterations in the social realm.

## Introduction

Puberty is a very important developmental period which is characterized by profound changes in an individual's brain (Romeo et al., 2002), physiology (Romeo, 2003, 2005), and behavior (Romeo, 2005). Brain regions like the medial prefrontal cortex (mPFC), hippocampus, and amygdala which are involved in learning, memory, emotion, cognition, and regulation of the hypothalamus-pituitary-adrenocortical (HPA) axis undergo extensive morphological and functional remodeling during this period (Spear, 2000; Giedd, 2004; Gogtay et al., 2004; Suzuki et al., 2005). The HPA axis is one of the main physiological stress systems and glucocorticoids (i.e., corticosterone or cortisol, depending on the species)—released by the adrenal glands—its final products. In addition to exerting a myriad of effects throughout the body, glucocorticoids are important modulators of brain structure and function (Joels and Vreugdenhil, 1998; De Kloet et al., 2008; Haller et al., 2008; Sandi, 2011).

The plasticity exhibited by the brain during the peripuberty period—comprising childhood and puberty—may make individuals more vulnerable to perturbations, such as stress. In fact, exposure to stress during this period is known to predispose to the development of psychopathologies such as depression and anxiety disorders later in life (Heim and Nemeroff, 2001; Watt et al., 2009). Animal models have recapitulated similar symptoms (Jacobson-Pick and Richter-Levin, 2010; Schmidt et al., 2011; Cordero et al., 2012; Márquez et al., 2013). In addition, exposure to fear or maltreatment during childhood and puberty increases the risk of developing violent behaviors in adulthood, as also shown in humans (Caspi et al., 2002; Jonson-Reid et al., 2010; Perepletchikova and Kaufman, 2010) and animals (Wommack and Delville, 2003; Veenema and Neumann, 2009; Cordero et al., 2012; Márquez et al., 2013).

These observations suggest that stress levels of corticosterone at peripuberty could play a key role in the programming of changes in subsequent behavior. Indeed, evidence indicating effectiveness for increased corticosterone in programing behavioral transitions during development has been reported. Thus, in male golden hamsters, cortisol treatment accelerates the transition from play fighting to adult aggression (Wommack and Delville, 2007), mimicking the effects induced by social subjugation stress applied over the same developmental period (Wommack and Delville, 2003).

Previous work from our laboratory has shown that exposing peripubertal rats to fear experiences (such as synthetic fox odor and exposure to an elevated platform) following an unpredictable schedule, on a range of 7 specific days across P28 to P42 (thus covering the equivalent to “childhood” and puberty periods of the rat; note that puberty in male Wistar rats typically takes place on P41 ± 1 day) leads to reduced sociability and increased aggression, and anxiety- and depression-like behaviors in adulthood (Cordero et al., 2012; Márquez et al., 2013). As expected, corticosterone levels are enhanced by stress exposure during the peripuberty period in this model (Márquez et al., 2013). Therefore, we hypothesized that administration of corticosterone following the same experimental schedule as for stress administration in our peripubertal paradigm (Cordero et al., 2012; Márquez et al., 2013) would mimic the alterations induced by peripubertal stress on adult behaviors, including social and anxiety- and depression-like behaviors. In addition, since HPA axis activity can also be regulated by early life experiences (Heim and Nemeroff, 2001; Heim et al., 2001; Rinne et al., 2002; Tarullo and Gunnar, 2006), we aimed to evaluate the impact of increasing corticosterone levels at peripuberty on the corticosterone response to stress exposure at adulthood. Finally, we also aimed at examining play behavior during adolescence to ascertain if potential changes in social behaviors identified at adulthood could also be observed shortly after treatment.

## Materials and methods

### Animals

Experimental subjects were the male offspring of Wistar Han rats purchased from Charles River Laboratories, France, and bred in our animal house. After weaning at PD21, male rats from different litters were mixed throughout the different home cages (three per cage). Equivalent numbers of animals from each litter were placed in the two experimental groups and siblings were avoided in the same home cage. All animals were kept in constant conditions of humidity and temperature (22 ± 1°C) with a 12-h light-dark cycle (lights on at 7:00 AM). Food and water were available *ad libitum*. All the procedures described were conducted in conformity with Swiss National Institutional Guidelines on Animal Experimentation, and approved through a license by the Swiss Cantonal Veterinary Office Committee for Animal Experimentation.

### Experimental design

**Figure 1:**
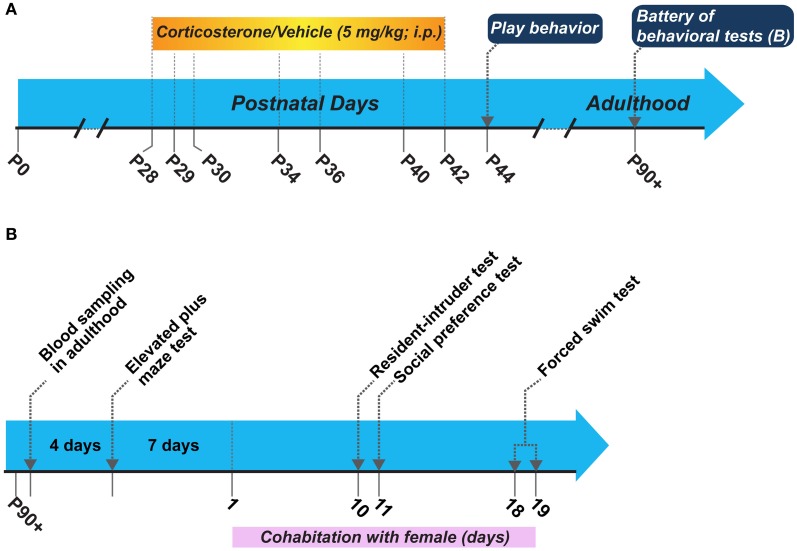
**(A)** Schematic of the general experimental design, and **(B)** Sequence of behavioral tests performed in adulthood. P, postnatal day.

The experimental schematic and the sequence of behavioral tests in adulthood is shown in Figure 1. We utilized two groups of animals for this study: (1) corticosterone injected and (2) vehicle injected. We administered corticosterone or vehicle following the same schedule as in our peripuberty stress paradigm described in (Toledo-Rodriguez and Sandi, 2011; Toledo-Rodriguez et al., 2012; Márquez et al., 2013) without actual stress exposure. We evaluated the play behavior in these animals during the early post-puberty period on P44 (note that puberty in male Wistar rats typically takes place on P41 ± 1 day) in their home cage and their behaviors in tests for anxiety-like behaviors [i.e., the elevated plus maze (EPM)], aggression [i.e., resident-intruder (RI) test], depression-like behaviors (i.e., the forced swim test), and motivation for social exploration (i.e., social exploration test) in adulthood. As a requirement to perform the RI tests, each of the male rats was put to cohabitate with a female of the same strain. The cohabitation started 7 days after the EPM and continued during 20 consecutive days. Thus, the RI test, the social preference test and the forced swim test were performed during this cohabitation period (Figure 1B). Before starting the battery of behavioral tests at adulthood and the cohabitation with the female, we exposed animals to novelty to measure their corticosterone responses. All behavioral scoring performed throughout the study was made by a researcher blinded to the treatment condition.

### Drug administration

Corticosterone was injected intraperitoneally (i.p.) as corticosterone–HBC (2-hydroxypropyl-β-cyclodextrin) complex (Sigma Chemical Co., Switzerland) at a dose of 5 mg/kg. This dose was selected because it was previously shown to mimic plasma steroid concentrations produced by substantial stress (Stein-Behrens et al., 1994; Venero et al., 1996). The corticosterone–HBC complex (7.8 mg/ml) was dissolved in saline (0.9% NaCl) and, thus, saline was used as the vehicle. Vehicle or corticosterone was administered on P28, P29, P30, P34, P36, P40, and P42 (injected volume ranged 65–150 μl depending on body weight).

### Corticosterone response after novelty

Immediately after 15 min exposure to a novel environment (a circular plastic container; 35 cm high, 25 cm diameter), blood samples were obtained by tail-nick (250–300 μl) within 2 min and, then, the rats were returned to their home cage. Animals from the same homecage were simultaneously tested in adjacent containers. The containers were cleaned with 1% acetic acid and dried properly before placing the animals. Data from one cage from the vehicle-treated group (three animals) had to be excluded from the analysis due to a sudden disturbance that occurred at the time the animals were tested.

### Plasma corticosterone

Blood samples were collected into ice-cold heparin capillary tubes (Sarsted, Switzerland). Plasma was obtained after blood centrifugation at 10,000 rpm for 4 min and it was stored at −20°C until analyses. Plasma corticosterone levels were measured by enzymatic immunoassay kit (Correlate-EIA from Assay Designs Inc., USA) according to supplier's recommendations.

### Play behavior

Play behavior was assessed on P44, 2 days after the last injection of vehicle or corticosterone. To stimulate play-fighting, home cages were changed before starting the recording. The recording was done for 10 min and the behavioral scoring was done off-line using computer software (clicker, EPFL, Switzerland). The duration of the following play-fighting behaviors was scored: pinning (i.e., keeping down), pouncing (i.e., rubbing the nape of the neck), jabbing (i.e., boxing), biting and kicking. Total duration of play fighting behavior was computed as the sum of the time spent in all those behaviors.

It should be noted that in order to allow our animals to develop under undisturbed conditions, in equivalent ways as in our peripuberty stress studies in which play behavior was not measured (Márquez et al., 2013), we did not perform standard measures of play behavior (Pellis and Pellis, 1990; Trezza and Vanderschuren, 2008; Veenema and Neumann, 2009) which might explain the lesser degree of play behavior observed in our study as compared to those. Most of the studies record play behavior on 5 weeks old rats, but we focused on P44 as our last injection was given on P42. Further, unlike other studies where rats are singly housed for few hours (Trezza and Vanderschuren, 2008) or a day (Veenema and Neumann, 2009) followed by an introduction of an intruder before recording play behavior, in order to avoid any unspecific behavioral effects that isolation during the juvenile period might induce on adulthood, we did our studies of play behavior with homecage mates without any former social isolation.

### Elevated plus maze

Anxiety levels were evaluated using the EPM test (Pellow and File, 1986). Briefly, the test consists of two opposing open arms (50 × 10 cm) perpendicular to two enclosed arms (50 × 10 × 50 cm) that extend from a central platform (10 × 10 cm), elevated 65 cm above the floor. The rats were placed individually on the central platform facing a closed arm and allowed to explore the maze for 5 min. Their behavior was monitored using a video camera and analyzed with a computerized tracking system (Ethovision 3.1.16, Noldus IT, The Netherlands). The percent time spent and the number (frequency) of entries in the center, open and closed arms were recorded. The entire apparatus was cleaned with 1% acetic acid solution and dried properly between each test.

### Resident-intruder test

Rats underwent the RI test at the age of 3.5 months. The RI protocol was adapted from Veenema et al. (2006) and followed the same conditions as in our previous peripuberty stress study (Márquez et al., 2013). Briefly, each rat was housed in an experimental cage (40 × 29 × 20 cm) together with a naive adult female Wistar rat. At the tenth day of the cohabitation with the female the RI test was performed (Figure 1B). Thirty minutes before the test, the female was removed from the resident's home-cage and was returned after the test was over. The test was carried out during the beginning of the dark cycle (between 19:30 and 21:30). The resident vehicle- or corticosterone-treated male rat was exposed in its homecage to a slightly smaller (5% of body weight difference), unfamiliar male Wistar rat for 30 min. Rats were exposed to a single RI test and intruders were used only once. The tests were videotaped and the behavioral scoring was done offline. The following parameters related to aggression were scored for residents: number of attacks, percent time spent of total aggressive behavior (sum of the percent of four aggressive behaviors: attack, lateral threat, offensive upright, and keep down). Percentage time of social non-aggressive behavior by the resident (hetero-grooming and sniffing the intruder) was measured as well.

### Social preference test

The social preference test was adapted from the protocol described by Crawley and collaborators to investigate social affiliation in male mice (Moy et al., 2004) and following the same conditions as in our previous peripuberty stress study (Márquez et al., 2013). The test was carried out in a rectangular, three-chambered gray opaque polycarbonate box (a center 20 × 35 × 35 cm; a left and a right compartment 30 × 35 × 35 cm). Dividing walls had retractable doorways allowing access to each chamber. Left and right compartment contained a central Plexiglas cylinder (15 cm diameter), transparent and with small holes, where either a social (unfamiliar juvenile rat around 34 days old) or a non-social stimulus (yellow plastic bottle) were placed. The cylinder permits visual, tactile, auditory and olfactory communication. The juvenile rats were first habituated to the three-chambered apparatus by placing them individually in the box within the Plexiglas cylinder for 10 min during the 3 consecutive days preceding the social test.

On the testing day, the experimental rat was first placed in the middle chamber and allowed to explore for 5 min. The doorways into the two side chambers were closed during this habituation phase. After the habituation period, the unfamiliar juvenile was placed in one of the side chambers and the object in the other side. The location of the juvenile and the object in the left vs. right side chamber was counterbalanced. Next, both doors to the side chambers were carefully removed and the subject rat was allowed to explore the entire apparatus for a 10-min session. The session was video-recorded and the time spent sniffing each cylinder was scored offline to evaluate the level of preference for the unfamiliar juvenile as compared to the object. The rats were considered to explore the object and the juvenile when their behavior complied with the following criteria: (1) when they were approaching the cylinders with their nose at a distance less than approximately 2 cm (2) when their nose was oriented toward the cylinders' contents (i.e., juvenile rat or object). These criteria of proximity and orientation were followed and quantified as sniffing behavior. The entire apparatus was cleaned with 1% acetic acid solution and dried properly between each test. Due to technical problems, three video files were lost and data from two vehicle-treated and one corticosterone-treated animals were lost.

### Forced swim test

Rats were submitted to a forced-swim test to evaluate depression-like behavior (Porsolt et al., 1977) and following the same conditions as in our previous peripuberty stress study (Márquez et al., 2013). Briefly, animals were individually placed in a plastic beaker (25 cm diameter, 46 cm deep) containing 30 cm of water (25°C) for 15 min. A second session was performed 24 h later for a 5-min test. Behavior was recorded with a video camera and the time spent immobile (making only those movements necessary to keep the snout above the water), was quantified offline.

### Statistical analysis

The SPSS 14.0 (SPSS, Chicago, IL) statistical package was used for the statistical analyses. Results are expressed as the mean ± standard error of the mean (SEM). Unpaired Student's *t*-tests were performed. If Levene's test for equality of variances was significant, equal variance was not assumed and the altered degrees of freedom were rounded to the nearest whole number. Values in graphs are represented as mean ± SEM. Results are considered to be statistically significant when the *p* value < 0.05.

## Results

### Play behavior during adolescence

**Figure 2:**
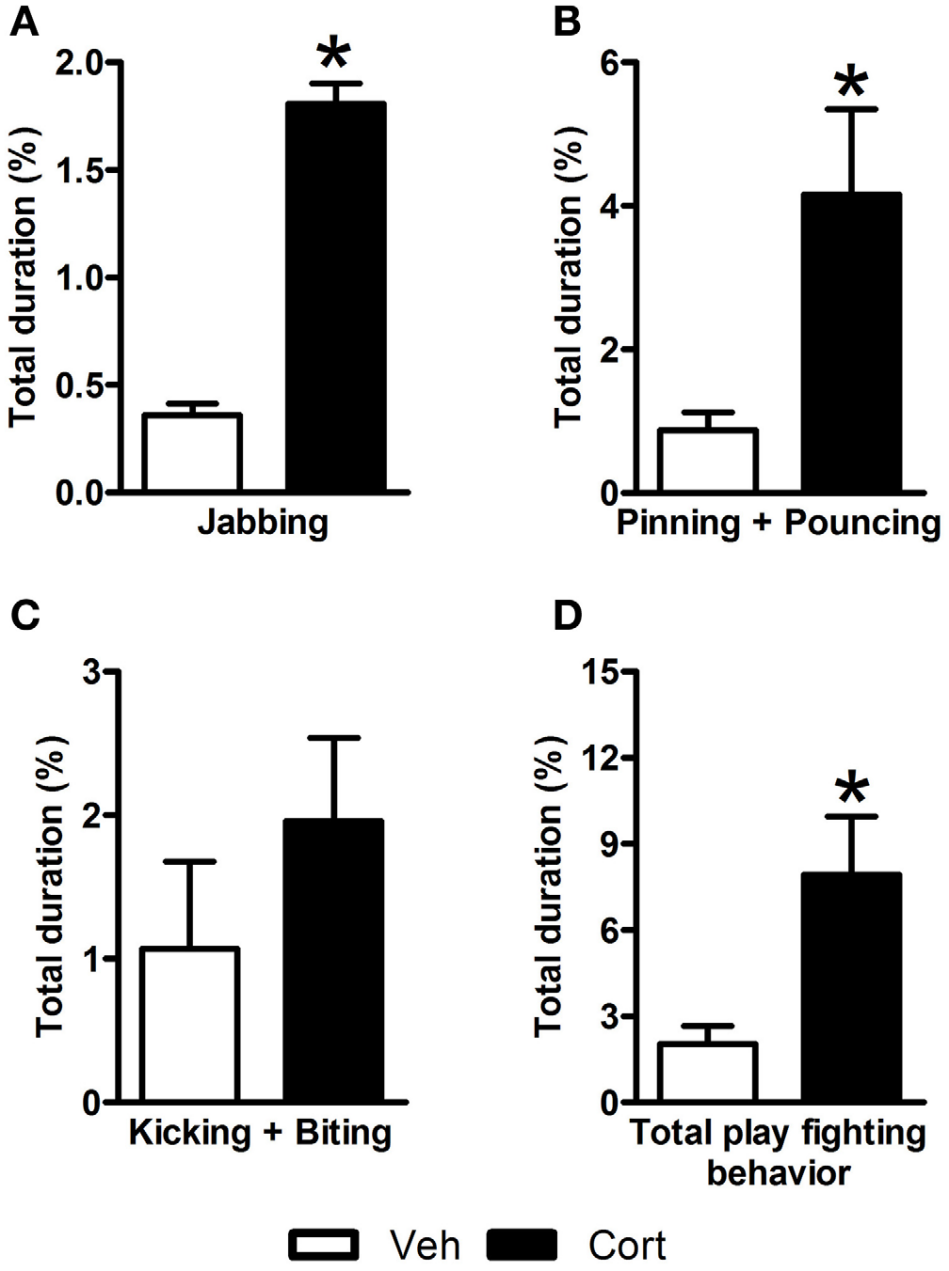
**Effect of corticosterone injections during peripuberty on play behavior. (A)** Percent duration of jabbing. **(B)** Percent duration of pinning and pouncing. **(C)** Percent duration of kicking and biting. **(D)** Percent duration of total play fighting behavior. The results are expressed as mean ± SEM; ^*^p < 0.05; *N* = 12/group.

In an attempt to examine the effect of corticosterone administration during the peripubertal period on the nature of social interactions of rats during adolescence, play behavior was observed on P44 (i.e., two days after the last injection). A Student *t*-test revealed higher duration of jabbing (Figure 2A: *t* = 4.725, *df* = 12, *p* < 0.05), pinning, and pouncing (Figure 2B: *t* = 2.702, *df* = 12, *p* < 0.05) and total play fighting behavior (Figure 2D: *t* = 3.281, *df* = 21, *p* < 0.05) in corticosterone-treated animals than in vehicle-treated ones. No statistical difference was observed between the two groups for a compound measure including kicking and biting (Figure 2C: *t* = 1.051, *df* = 21, n.s.). These data indicate that the administration of corticosterone during peripuberty increases play fighting behavior during the adolescence period.

### Plasma corticosterone in response to novelty

**Figure 3:**
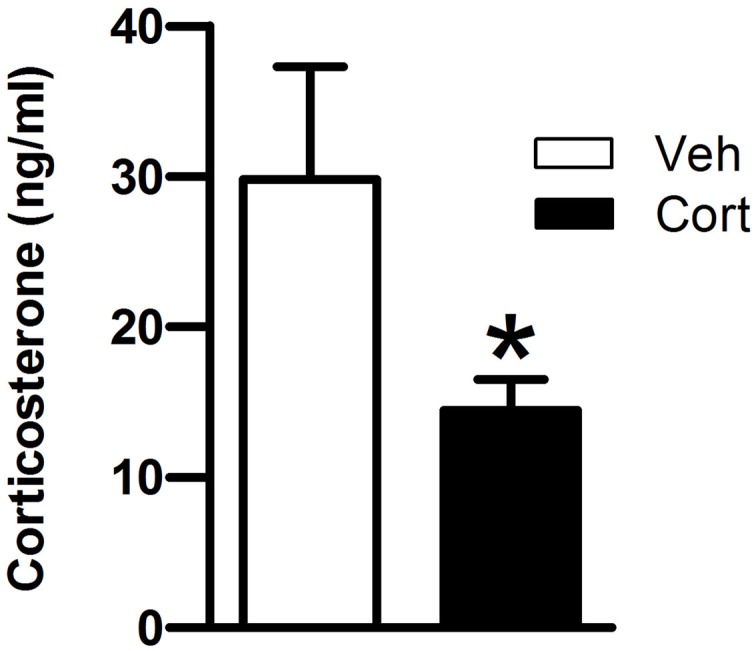
**Effect of corticosterone injections during peripuberty on the corticosterone response to a novel environment in adulthood.** Blood samples were taken immediately after a 15-min novelty exposure. The results are expressed as mean ± SEM; ^*^p < 0.05; *N* = 11 for Cort group and 9 for Veh group.

Then, we asked whether peripuberty corticosterone treatment would affect plasma corticosterone responses when exposing animals to a novel environment at adulthood. Data of one rat from the corticosterone-treated group was removed as the mean value for that animal was >3 standard deviations (SD) from its respective group mean. A Student *t*-test revealed a significant lower corticosterone level in the corticosterone-treated than in the vehicle-treated group (Figure 3: *t* = −2.160, *df* = 18, *p* < 0.05). This result indicates that exposure to increased corticosterone levels during peripuberty can lead to reduced corticosterone reactivity to mild stressors at adulthood.

### Anxiety-like behaviors

In order to examine the effect of peripuberty corticosterone administration on anxiety-like behaviors at adulthood, we subjected the animals to the EPM. No significant differences were observed between the groups in any of the parameters analyzed; i.e., the frequency of entries into and percent duration of time in the center, closed and open arms (all n.s.; Table 1). These results do not support the hypothesis that exposure to increased corticosterone levels during peripuberty affects animals' anxiety-like behaviors at adulthood.

**Table 1:** **Effect of corticosterone or vehicle administration during peripuberty on anxiety-like behaviors as assessed in the elevated plus maze in adulthood**.

**Elevated plus maze**	**Vehicle**			**Corticosterone**			***t***	***df***	***p***
**Variable**	**Mean**	***SEM***	***N***	**Mean**	***SEM***	***N***			
Frequency in center	16.83	1.66	12	16.83	1.38	12	0	22	n.s.
Frequency in closed arms	13.67	1.18	12	14.25	1.22	12	0.34	22	n.s.
Frequency in open arms	5.42	1.82	12	4.75	1.61	12	−0.27	22	n.s.
Percent time in center	23.34	2.44	12	22.91	1.57	12	−0.14	19	n.s.
Percent time in closed arms	68.5	4.57	12	69.64	2.57	12	0.21	17	n.s.
Percent time in open arms	6.68	1.89	12	6.04	1.67	12	−0.25	22	n.s.

No difference between groups was found in any of the parameters examined. Results are expressed as the mean and standard error of the mean (SEM). The statistical information [t-value, degree of freedom (df), and p-value] of unpaired Student t-tests are also provided in the table.

### Aggressive behaviors

**Figure 4:**
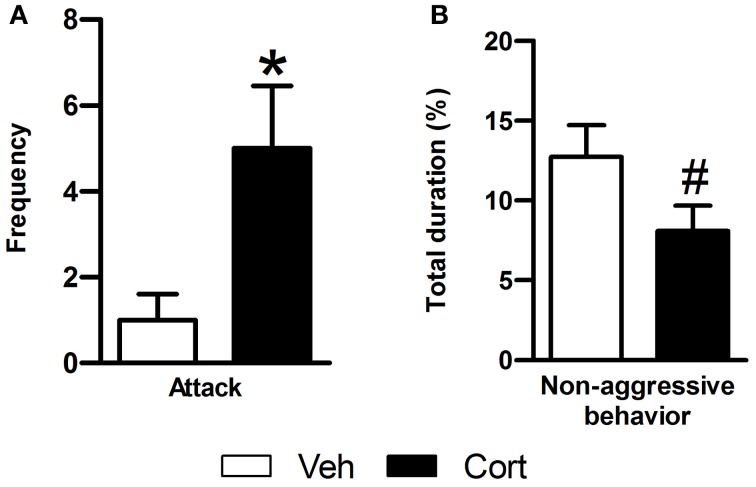
**Effect of corticosterone injections during peripuberty on aggressive behaviors in the resident intruder test in adulthood. (A)** Attack number. **(B)** Percent duration of non-aggressive behavior. The results are expressed as mean ± SEM; ^*^p < 0.05, ^#^*p* < 0.1; *N* = 11 for Veh and 12 for Cort group.

To evaluate the effect of peripuberty corticosterone administration on aggressive behaviors in adulthood, we performed a RI test. Data of animals with mean value >3 SDs or <3 SDs from its respective group mean were removed from the analyses. A Student *t*-test revealed a significant increase in the number of attacks in the corticosterone-treated group as compared to vehicle-treated animals (Figure 4A: *t* = 3.108, *df* = 17, *p* < 0.05). When non-aggressive social behaviors were analyzed, corticosterone-treated animals displayed a marginal non-significant reduction in these behaviors as compared to the vehicle-treated group (Figure 4B: *t* = −1.829, *df* = 21, *p* = 0.082). Thus, the results of this test indicate that exposure to increased corticosterone levels during peripuberty leads to higher aggressive behaviors in adult rats.

### Motivation for social exploration

**Figure 5:**
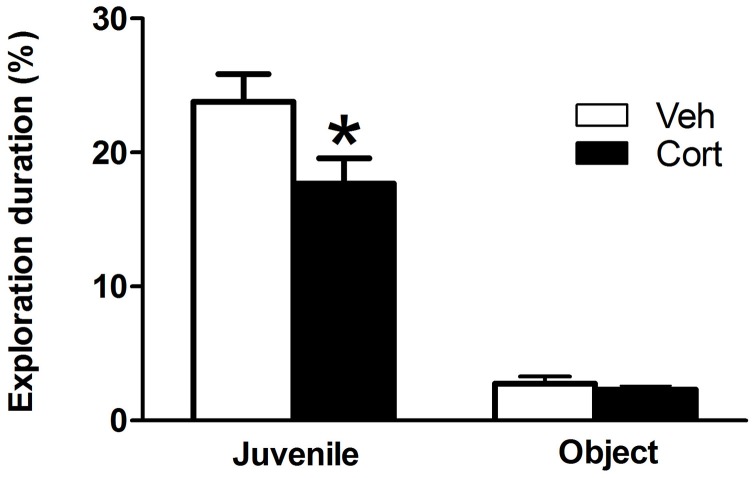
**Effect of corticosterone injections during peripuberty on social preference test in adulthood.** The graph shows the percent time of total juvenile exploration and percent time of total object exploration. The results are expressed as mean ± SEM; ^*^p < 0.05; *N* = 10 for Veh and 11 for Cort group.

To assess the effect of peripuberty corticosterone administration on sociability in adulthood, we performed a social preference test. A Student *t*-test revealed a significantly decreased percent time of juvenile exploration in the corticosterone-treated group as compared to the vehicle-treated group (Figure 5: *t* = −2.204, *df* = 19, *p* < 0.05). No significant difference was observed between groups for the percent time of object exploration (Figure 5: *t* = −0.675, *df* = 19, n.s). These results indicate that sub exposure to increased corticosterone levels during peripuberty leads to a specific reduction in the motivation to explore unfamiliar conspecifics.

### Depression-like behaviors

Depressive-like behavior in adulthood was assessed with the forced swim test. A Student *t*-test indicated that administration of corticosterone during puberty does not change depression-like responses in adulthood, as no statistical significant differences were observed between groups on the percent time floating on day 1 or day 2 (all n.s.; Table 2). Therefore, the results of this experiment do not support the hypothesis that elevated corticosterone levels at puberty render individuals more depressive-like when confronted to adversity at adulthood.

**Table 2:** **Effect of corticosterone or vehicle administration during peripuberty on depression-like behavior as evaluated in the forced swim test in adulthood**.

**Forced swim test**	**Vehicle**			**Corticosterone**			***t***	***df***	***p***
**Variable**	**Mean**	***SEM***	***N***	**Mean**	***SEM***	***N***			
Percent time floating (day 1)	62.31	2.84	12	57.16	3.64	12	−1.11	22	n.s.
Percent time floating (day 2)	52.78	3.5	12	45.99	4.86	12	−1.13	22	n.s.

No difference between groups was found in any of the parameters examined. Results are expressed as the mean and standard error of the mean (SEM). The statistical information [t-value, degree of freedom (df), and p-value] of unpaired Student t-tests are also provided in the table.

## Discussion

In this study, we show that corticosterone administration in male rats on scattered days across the peripuberty period leads to altered social behaviors—increased aggression and diminished social exploration—and reduced corticosterone reactivity to novelty in adulthood. Increased aggressive patterns in corticosterone-treated rats were already observed during play behavior at adolescence. However, this corticosterone treatment did not modify animals' responses at adulthood in tests for anxiety- and depression-like behaviors.

A main goal of our study was to compare the behavioral outcomes at adulthood of the peripuberty corticosterone treatment with the ones obtained in our former study in which we exposed animals to stress on the same developmental days (i.e., 7 specific days during the peripuberty period, including P28-P30, P34, P36, P40, and P42) (Márquez et al., 2013). Importantly, our current work did not aim at ascertaining whether stress-induced corticosterone mediates the effects of peripuberty stress but, instead, whether a corticosterone treatment given according to the same schedule as the peripuberty stress protocol would lead to similar outcomes. In order to address the role of stress-induced corticosterone elevations on the outcomes of peripuberty stress (Márquez et al., 2013), future studies will be aimed at either inhibiting corticosterone release or blocking its effects through specific corticosteroid receptor antagonists. Whereas we found similarities in the alterations induced by both procedures in the social domain (i.e., they both resulted in increased aggressive behaviors and diminished sociability), increased anxiety- and depression-like behaviors observed following peripuberty stress (Márquez et al., 2013) were not reproduced by peripuberty corticosterone treatment. The discordance on the effects of both peripuberty treatments on measures of anxiety- and depression-like behaviors at adulthood might be due a variety of factors differing between these procedures, from differences in the actual corticosterone levels achieved by each procedure, to differences in the number of (neuro)physiological stress mediators and type of emotional processing that each of them activates. The latter might be particularly important for emotional programming of the developing brain. Importantly, in the peripuberty stress protocol, in addition to the activation of the HPA axis with the resulting increase in corticosterone levels on each peripuberty stress day, stress consists on exposing animals to two types of fear-induction experiences (elevated platform and predator odor), each one of 25 min duration, and depending on the day including one or two subsequent presentations in an unpredictable manner. Therefore, the stress protocol elicits strong innate fear responses, which are not presented with the corticosterone treatment, and that can be instrumental for the programming a personality with a bias toward anxiety- and depression-like behaviors. However, a note of caution should be added as we did not measure emotional responses in our animals during or immediately after the corticosterone administration protocol and, thus, we cannot discard altered emotionality in the aftermath of corticosterone injections. Future experiments should be addressed to directly examine whether corticosterone and emotional responsiveness is already affected by corticosterone injections at peripuberty and whether potential changes in these responses are linked with the behavioral outcomes of the treatment observed at adulthood.

Interestingly, Jacobson-Pick and Richter-Levin (2010) showed that the immediate emotional reaction in terms of exploration and anxiety-like responses induced by acute corticosterone treatment in rats differs when the steroid is given during the prepuberty period and at adulthood. Whereas when the treatment was given at adulthood (P60), animals displayed an anxious behavioral pattern, animals treated at prepuberty (P29) showed increased activity and a behavioral pattern (i.e., more activity in the center of the open field and in the open arms of the EPM) that in classical terms would be interpreted as reduced anxiety (Jacobson-Pick and Richter-Levin, 2010). Accordingly, given the “anxiolytic-like” effect elicited by corticosterone treatment when administered during the peripuberty period, it might not be surprising that such treatment, as observed in the present study, does not “program” individuals' behaviors toward high emotionality. Arguably, such programming effects of glucocorticoids might require the coupling with fearful or highly emotional experiences. In our work, anxiety- and depression-like behaviors at adulthood are observed in the peripuberty stress in which corticosterone responses are paired with activation of fear circuits (Márquez et al., 2013). That these circuits are affected by peripuberty stress is indicated by the increase metabolic rates displayed by stressed animals in hippocampus, basal amygdala and cingulate cortices when exposed to fear cues shortly after the end of the stress protocol (Toledo-Rodriguez et al., 2012). We hypothesize that corticosterone elevation due to the injection protocol would be acting in a non-aroused brain and, thus, having different programming effects than the peripuberty stress protocol. One way to test this hypothesis would be to expose animals to mild fearful experiences at puberty (ideally not triggering on their own a facilitation of anxiety- and depression-like behaviors at adulthood) and to give corticosterone injections paired with those experiences.

Whereas the effectiveness of different stressful experiences given in rodents around peripuberty to increase aggressive (Cordero et al., 2012; Márquez et al., 2013), as well as anxiety- (Schmidt et al., 2007; McCormick et al., 2008; Bazak et al., 2009; Lukkes et al., 2009; Ros-Simo and Valverde, 2012) and depression-like (Tsoory et al., 2007; Hong et al., 2012) behaviors has been largely documented in the literature, to our knowledge there are virtually no studies that have examined the long-term behavioral impact of peripuberty corticosterone treatments. Indirect evidence for a potential role of enhanced glucocorticoids at peripuberty to exert an impact on subsequent behaviors can be found in tangentially related studies. In golden hamsters, for example, glucocorticoid treatment (i.e., either the synthetic glucocorticoid dexamethasone or cortisol) given during the prepuberty period (from P31 to P36) was shown to facilitate the transition from play fighting to adult aggression (Wommack et al., 2005; Wommack and Delville, 2007), an effect that was blocked by a co-treatment with a glucocorticoid receptor antagonist (Wommack and Delville, 2007). Importantly, in this model glucocorticoid treatment produced the same effects in accelerating the transition in the nature of the fighting behaviors from play fighting to adult aggression as observed in animals submitted to repeated social defeat during prepuberty (Wommack and Delville, 2003; Wommack et al., 2004). Although the neuroendocrine development throughout the peripuberty period and the nature of play fighting interactions differ in golden hamsters and rats (Wommack and Delville, 2007), and therefore it is not possible to directly refer these findings in hamsters to our rat models, these studies highlight a key role for glucocorticoids during the peripuberty period on the regulation of fighting behaviors. Another example of the programing power of enhanced glucocorticoid levels was reported in rats: corticosterone treatment given daily during the pre-puberty period (from P24 to P39) in combination with a psychostimulant drug was shown to lead to a sensitization to the drug when administered again in adulthood (Juarez and Vazquez-Cortes, 2010). More abundant are the studies that, instead of testing their long-term programming, confirmed the efficiency of chronic corticosterone treatments (typically involving very high doses for at least 3 weeks, while milder conditions were inefficient) given in adulthood to increased anxiety-like and depression-like behaviors (Johnson et al., 2006; Zhao et al., 2008; David et al., 2009; Marks et al., 2009).

Regarding the long-term effects of our peripuberty corticosterone treatment on adult aggression, it is important to note that these animals already showed increased aggressive play fighting when play behavior was scored during the early post-puberty period on P44 (but note that the measurement of play behavior in our study differs from standard procedures in the literature; see details in the Materials and Methods section). Therefore, although we cannot discard that the treatment might have induced as well a latent change on a behavioral program only emerging at adulthood, the increases in play fighting suggest that the enduring effects of corticosterone in the social domain (increased aggression, reduced social exploration) might be the result of a more immediate action of the treatment on the nature of social interactions established in the homecage. Such change in the dynamic social interactions during the peripuberty period could have influenced the neurodevelopmental trajectory of these animals. Although we do not have data on play behavior following our peripuberty stress protocol and, therefore, we cannot compare this behavior between the two protocols, evidence from another stress model based on maternal separation and leading to increased aggressive behavior in adulthood (Veenema et al., 2006) as well as in play fighting during peripuberty (Veenema and Neumann, 2009), supports the view that long-term social changes related to early life stress or corticosterone treatment might be anchored on sustained behavioral changes already detected in the earlier aftermath of stress. That increasing glucocorticoid levels can have an immediate effect on increasing aggressive behaviors has been elegantly illustrated in adult rats (Haller et al., 1997; Mikics et al., 2004, 2007). Interestingly, the heightening of aggressive behaviors by corticosterone under social challenge was found to be maintained in future exposures to aggressive contests (Mikics et al., 2007), supporting the view that long-term effects of increased corticosterone on aggressive behaviors can be the result of a “clamping” (and thus programming) effect exerted by the steroid on its immediate behavioral action.

Therefore, we hypothesize that one important reason why the corticosterone treatment reproduces some effects of peripuberty stress (i.e., the ones in the social domain), but not others (i.e., the ones in the emotional domain) is that corticosterone injections were not coupled with any specific emotional challenge but with animal's social interactions with their cagemates (note that immediately after each injection, animals were returned to their homecages). Thus, we are putting forward the hypothesis that corticosterone's programming actions should be envisioned in the context of the actual state of activated emotion and information processing brain circuits co-occurring with high glucocorticoid levels. A way to probe this hypothesis in the context of the current findings would be to briefly prevent social interactions for a few hours following each corticosterone injection and comparing the results in the social domain with the ones reported here. An alternative hypothesis is that other systems and mechanisms in addition to and/or instead increased corticosterone are involved in the long-term actions of peripuberty stress. Although addressing this question is beyond the scope of the present study, its plausibility is supported by several studies linking early life stress with emotional alterations in adulthood and pointing at the deregulation of a variety of potential non-exclusive mediating systems, including the limbic monoaminergic systems (Watt et al., 2009; Diamantopoulou et al., 2012), the corticotropin-releasing factor (CRF) system (Lukkes et al., 2009), the hypothalamic vasopressinergic system (Veenema et al., 2006), and hippocampal brain derived neurotrophic factor (BDNF) (Bazak et al., 2009; Coppens et al., 2011). Our ongoing work implicates the CRF system among such mediating mechanisms.

Finally, we also found that corticosterone administration during puberty leads to a reduced corticosterone response to novelty at adulthood. Although we have no equivalent data from our peripuberty stress protocol to compare the outcome of the two treatments, several studies have shown that stress or trauma exposure during peripuberty is associated with lower cortisol in adulthood in both, humans (Hamilton et al., 2008) and animals (Bazak et al., 2009). In humans, blunted cortisol responses have been repeatedly reported in posttraumatic stress disorder (PTSD) patients (Yehuda et al., 1990, 1995, 1996; Resnick et al., 1995; Golier and Yehuda, 1998), and progressive attenuation of cortisol across age has been reported in victims of childhood sexual abuse (Trickett et al., 2010). Likewise, in rodents, exposure to predator scent stress in juvenile rats is associated with reduced basal corticosterone in adulthood (Bazak et al., 2009). Low levels of glucocorticoids may reflect alterations of the HPA-axis, convening increase vulnerability for stress-related disorders. Importantly, in rats, adult-like corticosterone stress responses (i.e., diminution of the heightened hormonal stress reactivity found during early prepuberty) have been described to change between P30 and P40 (Foilb et al., 2011), a time window that might course with increased vulnerability and that comprises the central part of our corticosterone injection protocol.

Taken together, our findings show that increasing corticosterone levels on scattered days during the peripuberty period has immediate and long-lasting effects in the social domain, in similar ways as following peripuberty stress. However, corticosterone treatment did not reproduce the long-lasting effects of peripuberty stress in the emotional domain. These data suggest that increased glucocorticoid levels during peripuberty might be instrumental to program alterations in social life observed following stress. They also open the possibility that individual differences on the degree of HPA axis activation during peripuberty might be central to defining differences in vulnerability to develop psychopathology.

### Conflict of interest statement

The authors declare that the research was conducted in the absence of any commercial or financial relationships that could be construed as a potential conflict of interest.
